# Autophagy blockade and lysosomal membrane permeabilization contribute to lead-induced nephrotoxicity in primary rat proximal tubular cells

**DOI:** 10.1038/cddis.2017.262

**Published:** 2017-06-08

**Authors:** Xiang-Bin Song, Gang Liu, Fei Liu, Zhen-Gui Yan, Zhen-Yong Wang, Zong-Ping Liu, Lin Wang

**Affiliations:** 1 College of Animal Science and Veterinary Medicine, Shandong Agricultural University, Tai'an, China; 2 College of Veterinary Medicine, Yangzhou University, Yangzhou, China; 3 Jiangsu Co-innovation Center for Prevention and Control of Important Animal Infectious Diseases and Zoonoses, Yangzhou, China

## Abstract

Lead (Pb) is a known nephrotoxicant that causes damage to proximal tubular cells. Autophagy has an important protective role in various renal injuries, but the role of autophagy in Pb-elicited nephrotoxicity remains largely unknown. In this study, Pb promoted the accumulation of autophagosomes in primary rat proximal tubular (rPT) cells, and subsequent findings revealed that this autophagosome accumulation was caused by the inhibition of autophagic flux. Moreover, Pb exposure did not affect the autophagosome–lysosome fusion in rPT cells. Next, we found that Pb caused lysosomal alkalinization, may be through suppression of two V-ATPase subunits. Simultaneously, Pb inhibited lysosomal degradation capacity by affecting the maturation of cathepsin B (CTSB) and cathepsin D (CTSD). Furthermore, translocation of CTSB and CTSD from lysosome to cytoplasm was observed in this study, suggesting that lysosomal membrane permeabilization (LMP) occurred in Pb-exposed rPT cells. Meanwhile, Pb-induced caspase-3 activation and apoptosis were significantly but not completely inhibited by CTSB inhibitor (CA 074) and CTSD inhibitor (pepstatin A), respectively, demonstrating that LMP-induced lysosomal enzyme release was involved in Pb-induced apoptosis in rPT cells. In conclusion, Pb-mediated autophagy blockade in rPT cells is attributed to the impairment of lysosomal function. Both inhibition of autophagic flux and LMP-mediated apoptosis contribute to Pb-induced nephrotoxicity in rPT cells.

Lead (Pb) is one of the most abundant toxic heavy metals in the environment which causes a broad range of biochemical, physiological and behavioral dysfunctions in human beings.^1^ Globally, Pb exposure is ubiquitous and routes of Pb exposure include inhalation of Pb-contaminated dust particles or aerosols, and ingestion of Pb-contaminated food or water.^1, 2^ The persistence of Pb in humans and its associated health risk is a matter of serious concern and a global issue. Pb is also a cumulative toxicant that is stored in the body for a long term, which exerts potent toxic effects on different tissues.^3^ Kidney is one of the most sensitive target organs for Pb toxicity, while the proximal tubule is the major site of Pb-induced renal injury.^4^ However, proximal tubular cells were less applied in the studies of Pb-induced nephrotoxicity. Primary cultures can better represent the live tissue than permanent cell lines, which are ideal for *in vitro* toxicity studies. Thus, primary rPT cells were established to elucidate low-level Pb-induced nephrotoxicity in this study.

Autophagy is a highly dynamic multi-step biological process which maintains cellular homeostasis via the degradation and recycling of damaged organelles, misfolded proteins, and long-lived macromolecules in lysosomes.^5^ Previous studies have demonstrated that basal autophagy is vital for normal proximal tubule function, and genetic or pharmacologic blockade of autophagy strongly enhanced acute kidney injury induced by cisplatin or ischemia-reperfusion.^6, 7, 8, 9^ Moreover, report by Lv *et al.*^10^ and Sui *et al.*^11^ showed that Pb promoted the autophagy in cultured osteoblasts and cardiofibroblasts, respectively. By contrast, our research group has recently found that 0.5 *μ*M Pb treatment for 12 h blocked the autophagic flux in rPT cells,^12^ while the underlying molecular mechanism of impaired autophagic flux during Pb exposure remains to be elucidated.

Lysosomes are acidic organelles that contain various hydrolytic enzymes, which serve as cellular recycling centers for cargos received mainly from autophagy and endocytosis. Normal lysosomal degradation function is crucial to maintain cellular homeostasis and enable cell survival in the physiological state.^13, 14^ Due to its high hydrolase content, lysosomes are potentially harmful to the cell when damage occurs to the lysosomal membrane to induce lysosomal membrane permeabilization (LMP).^14^ LMP has been shown to cause the release of cathepsins and other hydrolases from the lysosomal lumen to the cytosol, which is a critical step in lysosome-mediated apoptosis.^13^ We have previously demonstrated that the apoptotic death was the chief mechanism in low-dose (0-1.0 *μ*M) Pb-induced nephrotoxicity in rPT cells,^15^ which enable us to think whether LMP is involved in Pb-induced apoptosis in rPT cells. Based on the previous studies, this study will offer further evidences to elucidate the possible interaction mechanism between autophagy inhibition, impairment of lysosomal function and apoptosis in Pb-exposed rPT cells.

## Results

### Enhanced expression of autophagic marker LC3-II in Pb-exposed rPT cells

**Figure 1:**
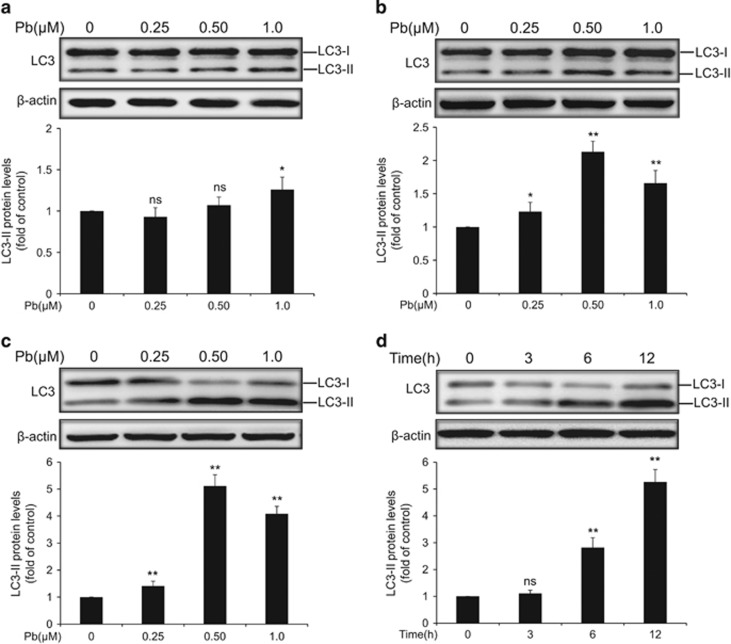
Expression of the autophagy marker protein LC3-II in Pb-exposed rPT cells. Cells were incubated with increasing doses of Pb for 3 h (**a**), 6 h (**b**) and 12 h (**c**) to assess the protein levels of LC3-II. (**d**) Cells were treated with 0.5 *μ*M Pb for different time periods to determine the protein levels of LC3-II. Upper panels: representative western blot images; lower panels: quantitative analysis of protein levels (mean±S.E.M., *n*=4). *ns*, not significant, *P<0.05, **P<0.01, as compared with control

Immunoblot analysis of endogenous LC3-II has been widely used to reflect the progression of autophagy. Firstly, protein levels of LC3-II in rPT cells treated with Pb (0.25, 0.5 and 1 *μ*M) for 3 h (Figure 1a), 6 h (Figure 1b) and 12 h (Figure 1c) was detected to investigate the effect of Pb exposure on autophagy, respectively. After 3 h treatment, only 1 *μ*M Pb significantly elevated the LC3-II protein levels (Figure 1a). Significant differences were observed in the LC3-II protein levels after exposed to Pb (0.25, 0.5 and 1 *μ*M) for 6 h (Figure 1b) and 12 h (Figure 1c), respectively; but 0.5 *μ*M Pb resulted in a somewhat greater increase in LC3-II level than 1 *μ*M Pb at these two time points. Likewise, there was a time-dependent enhancement of LC3-II protein level in 0.5 *μ*M Pb-treated cells (Figure 1d). Thus, 0.5 *μ*M Pb and 12 h exposure time were selected in subsequent experiments.

### Autophagic flux was impaired by Pb treatment in rPT cells

**Figure 2:**
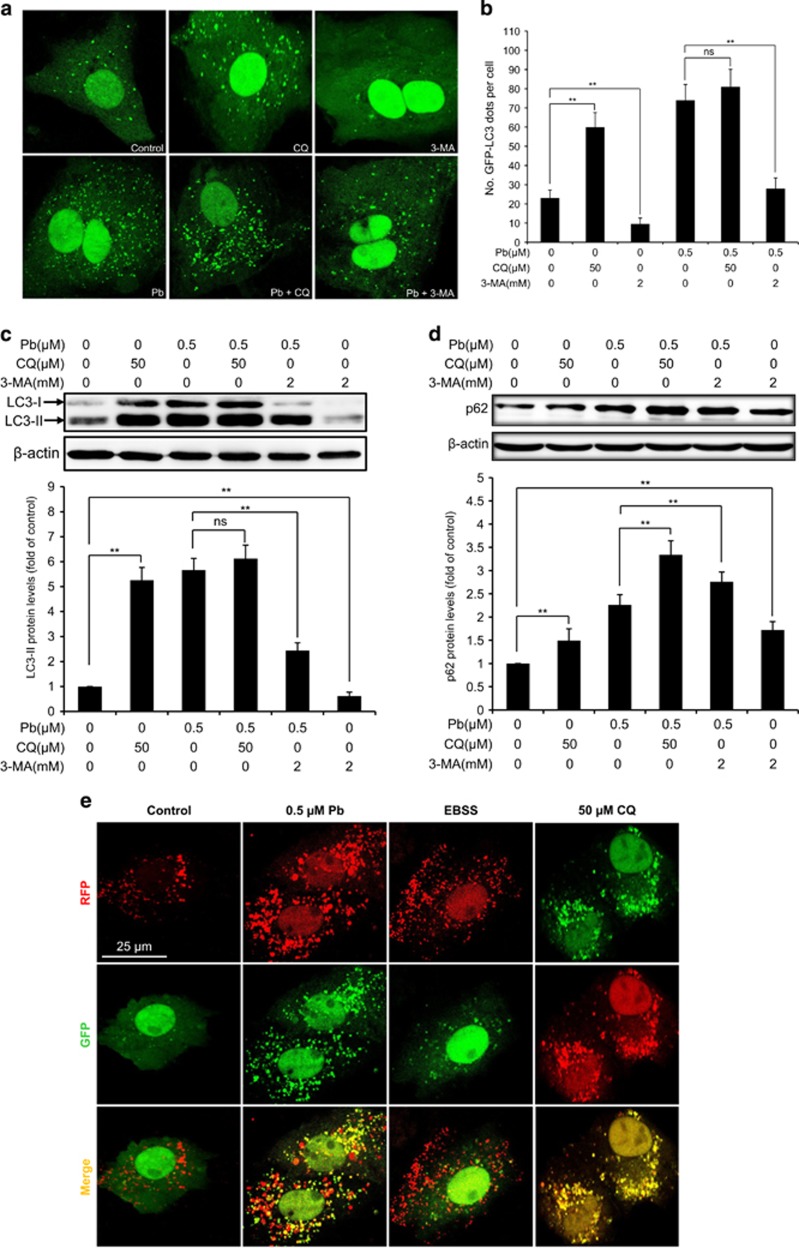
Inhibition of autophagic flux was revealed in Pb-exposed rPT cells. (**a** and **b**) Cells were transiently transfected with GFP-LC3 plasmid, then treated with 0.5 *μ*M Pb for 12 h in the presence or absence of CQ (50 *μ*M, for the last three hours), or 3-MA (2 mM, for the entire course). Representative confocal images are present in **a** and the number of GFP-LC3 dots per cell (**b**) was quantified (mean±S.E.M., *n*=4). (**c** and **d**) Cells were treated with 0.5 *μ*M Pb for 12 h in the presence or absence of 50 *μ*M CQ (for the last three hours), or 2 mM 3-MA (for the entire course); then harvested and analyzed for protein levels of LC3 (**c**) or p62 (**d**) using western blotting. Upper panels: representative western blot images; lower panels: quantitative western blot analysis (mean±S.E.M., *n*=4). (**e**) Cells were transiently transfected with GFP-RFP-LC3 plasmid, treated with 0.5 *μ*M Pb for 12 h or incubated with EBSS medium for 2 h, or treated with 50 *μ*M CQ for 3 h. Puncta showing both green and red fluorescence (indicated as yellow), or showing only the red fluorescence are shown in the representative confocal images of four groups. *ns*, not significant, **P<0.01

Although the amount of LC3-II correlates with the number of autophagosomes, increased numbers of autophagosomes can be associated either with increased autophagosomes synthesis or decreased autophagosomes turnover.^16^ To distinguish these two possibilities, we assessed the autophagic flux. First, the effect of Pb exposure on the autophagosome formation in the presence of 3-MA or CQ (two well-defined autophagy inhibitors) was assessed using transient transfection method (Figures 2a and b). Co-incubation of Pb with 3-MA, which blocks the upstream steps of autophagy,^17^ markedly reduced Pb-induced GFP-LC3 puncta accumulation. However, co-administration of Pb with CQ, which blocks the downstream steps of autophagy,^17^ did not cause a significant increase in GFP-LC3 puncta formation compared with Pb treatment alone, indicating that Pb did not stimulate autophagic flux in rPT cells. Consistent with this finding, western blot analysis showed that Pb-elevated LC3-II protein level was significantly reduced by 3-MA treatment, but not affected by CQ treatment (Figure 2c). Additionally, Pb treatment significantly promoted the accumulation of autophagic substrate p62, which further elevated by the addition of CQ or 3-MA (Figure 2d), indicating that the increased autophagosomes was due to impaired autophagosome clearance instead of increased formation.

Finally, we utilized a valuable tool for examining autophagy flux, the tandem RFP-GFP-LC3 construct. In normal condition, the LC3-II positive autophagosomes are labeled with yellow (GFP and RFP signals), and after fusion with lysosomes, autolysosomes are shown as red only puncta (GFP is more rapidly quenched than RFP by low lysosomal pH).^5, 18^ Compared with the control group, starvation (by culturing rPT cells in EBSS) greatly induced the increase of both yellow and red only puncta, while CQ significantly promoted the autophagosomes accumulation (yellow puncta). Also, Pb markedly promoted the accumulation of yellow puncta only, further confirming that autophagy flux was blocked (Figure 2e). Taken together, these findings strongly indicated that autophagic flux was impaired in Pb-exposed rPT cells.

### Pb did not inhibit fusion between autophagosomes and lysosomes in rPT cells

**Figure 3:**
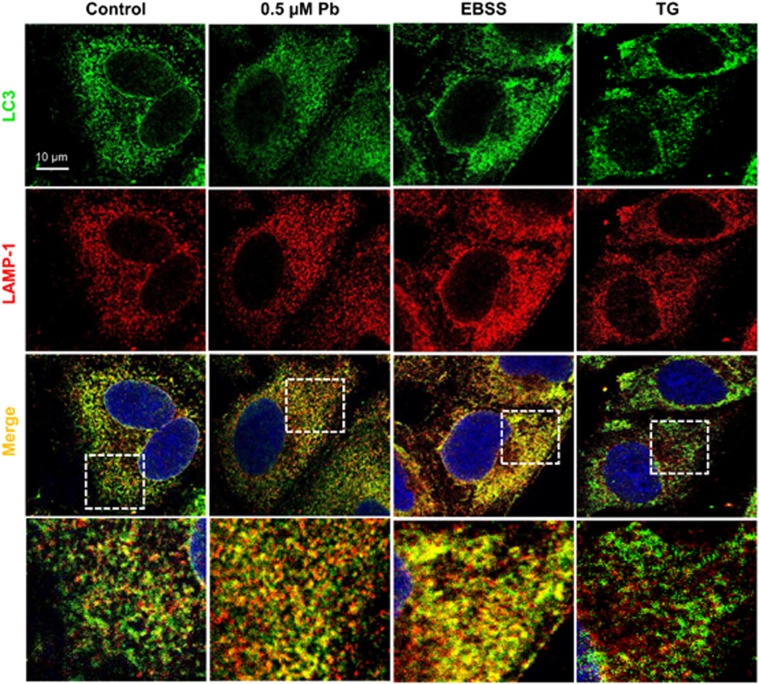
Pb enhanced autophagosome–lysosome fusion in rPT cells. Cells were treated with 0.5 *μ*M Pb or 1 *μ*M TG (negative control) for 12 h or incubated with EBSS medium for 2 h (positive control), then successively immunostained with LC3, LAMP-1 and nuclei were stained with DAPI. Representative confocal images are shown and panels on the bottom are higher-magnification images of the cropped regions

One possible explanation for the autophagy blockage in Pb-exposed rPT cells was due to the defective autophagosome–lysosome fusion. In this study, we used confocal microscopy to check for autolysosome formation (a complex structure of autophagosome fused with lysosome) by double labeling rPT cells with endogeneous LC3 staining (a marker of autophagosome) and LAMP-1 (a marker of lysosome). As shown in Figure 3, the LC3-positive autophagosome (green) were well colocalized with LAMP-1-positive lysosome (red) to form autolysosome (yellow) in rPT cells treated with 0.5 *μ*M Pb for 12 h compared with control cells, similar to the effect of starvation (2 h incubation in EBSS) (positive control). By contrast, TG treatment (negative control), which blocks the autophagosome–lysosome fusion,^19^ produced a significant reduction in colocalization of LC3 puncta and LAMP-1-positive compartments. Thus, data in Figure 3 clearly indicated that Pb did not block autophagosome–lysosome fusion.

### Pb caused lysosomal alkalinization in rPT cells

**Figure 4:**
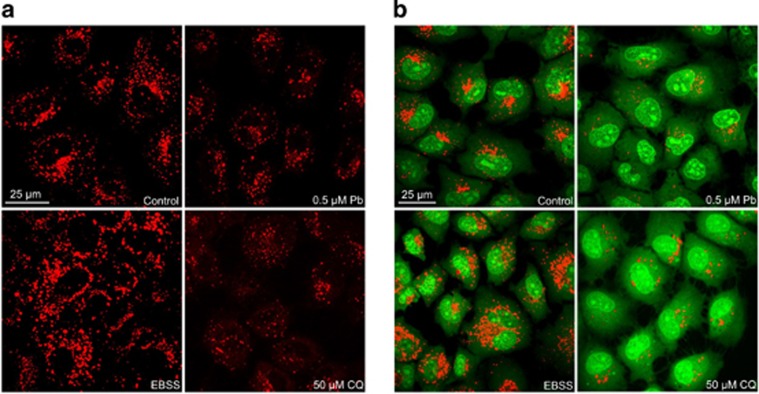
Pb affected the lysosome acidity in rPT cells. Cells were incubated with 0.5 *μ*M Pb for 12 h or 50 *μ*M CQ (negative control) for 3 h, or EBSS medium (positive control) for 2 h. After the indicated treatments, cells were stained with 100 nM Lyso-Tracker Red at 37 °C for 30 min (**a**) or 5 *μ*g/ml acridine orange at 37 °C for 30 min (**b**). Slides were viewed using a scanning confocal microscope

We then assessed whether Pb-induced autophagic flux impairment was due to the inhibition of lysosomal function. Two sensitive lysosomotropic pH probes were applied to evaluate the effect of Pb on lysosomal pH in rPT cells (Figure 4). Firstly, Lyso-Tracker Red (LTR) manifests red fluorescence in a pH-dependent manner in the lysosome and the increased staining indicates the reduced lysosomal pH.^20^ As shown in Figure 4a, starvation (2 h incubation in EBSS) enhanced lysosomal acidification (increased LTR staining) while CQ neutralized lysosomal pH (disappearance of the red fluorescence of LTR) compared with the control cells, confirming the effect of positive and negative control in this assay. Given this, Pb significantly abolished the LTR staining due to abolished lysosomal acidification in rPT cells. Secondly, AO staining presents green fluorescence in the cytosol but red fluorescence when it is accumulated in the acidic compartments due to its being highly protonated.^20^ Thus, decrease in granular red fluorescence with increase in diffuse green fluorescence implies the elevated lysosomal pH. Data in Figure 4b further verified that Pb caused lysosomal alkalinization in rPT cells.

### Effects of Pb on the V-ATPase subunits expression in lysosomes of rPT cells

**Figure 5:**
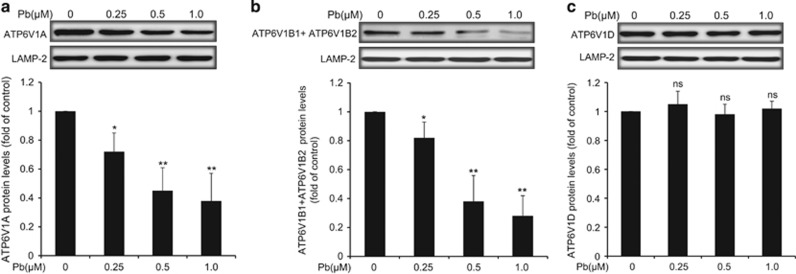
Effect of Pb on protein levels of three V-ATPase subunits in isolated lysosomes of rPT cells. Cells were treated with Pb (0.25, 0.5 and 1 *μ*M) for 12 h, then fractionated into the lysosomal lysis to assess the protein levels of ATP6V1A (**a**), ATP6V1B1+ATP6V1B2 (**b**) and ATP6V1D (**c**). Upper panels represent western blot images; lower panels represent quantitative analysis of protein levels (mean±S.E.M., *n*=4). *ns*, not significant, *P<0.05, **P<0.01, as compared with control

Since lysosome acidification is partially correlated with the activity of V-ATPase,^21^ changes in protein levels of three V-ATPase subunits located in lysosomes of rPT cells were assessed in this study. Compared with the control group, there was a dose-dependent decrease in ATP6V1A (Figure 5a) and ATP6V1B1+ATP6V1B2 (Figure 5b) protein levels in Pb-exposed cells. Also, there was no significant difference in ATP6V1D protein level between the control and Pb group (Figure 5c). These data indicated that suppression of two V-ATPase subunits (ATP6V1A, ATP6V1B1+ATP6V1B2) may be involved in Pb-induced lysosomal alkalinization.

### Pb suppressed lysosomal degradation of rPT cells

**Figure 6:**
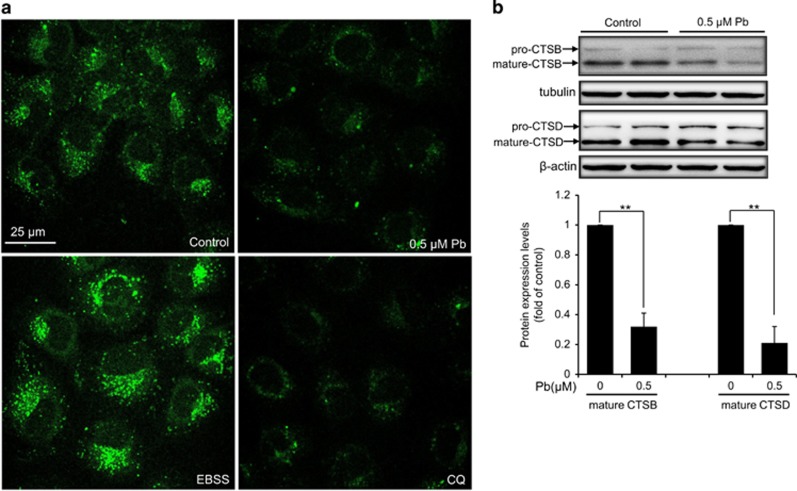
Pb attenuated the proteolytic activity in lysosomes of rPT cells. (**a**) Cells were pre-incubated with 10 *μ*g/ml DQ-BSA Green for 12 h, then refreshed the medium and treated with 0.5 *μ*M Pb for 12 h, 50 *μ*M CQ (negative control) for 3 h, or EBSS medium (positive control) for 2 h. Images were obtained by confocal microscopy. (**b**) Cells were treated with 0.5 *μ*M Pb for 12 h, then harvested to assess the protein levels of CTSB and CTSD using the whole-cell lysates. Upper panel represent western blot image; lower panel represent quantitative analysis of protein levels (mean±S.E.M., *n*=4). **P<0.01

DQ-BSA dequenching analysis, a lysosome-specific degradation assay, was chosen to investigate the effect of Pb on lysosomal degradation capacity of rPT cells. Upon proteolytic cleavage (normal lysosomal conditions), nonquenched protein fragments were released and became highly fluorescent.^22^ As shown in Figure 6a, DQ-BSA was efficiently cleaved during starvation (EBSS, 2 h) while treatment of CQ (a lysosomal inhibitor), as well as Pb exposure caused a significant reduction of DQ-BSA-related green fluorescence, confirming that Pb inhibited lysosomal degradation capacity of rPT cells.

Cysteine protease cathepsin B (CTSB) and aspartic protease cathepsin D (CTSD) are the most abundant lysosomal proteases.^23^ To examine how Pb might affect the lysosomal degradation, intracellualr protein levels of CTSB and CTSD in Pb-exposed rPT cells were assessed by western blotting analysis. Consistently, Pb significantly impaired the maturation of CTSB and CTSD (Figure 6b). Taken together, data in Figure 6 indicated that Pb inhibited lysosomal degradation capacity by affecting the maturation of selected proteases.

### Pb-induced LMP in rPT cells

**Figure 7:**
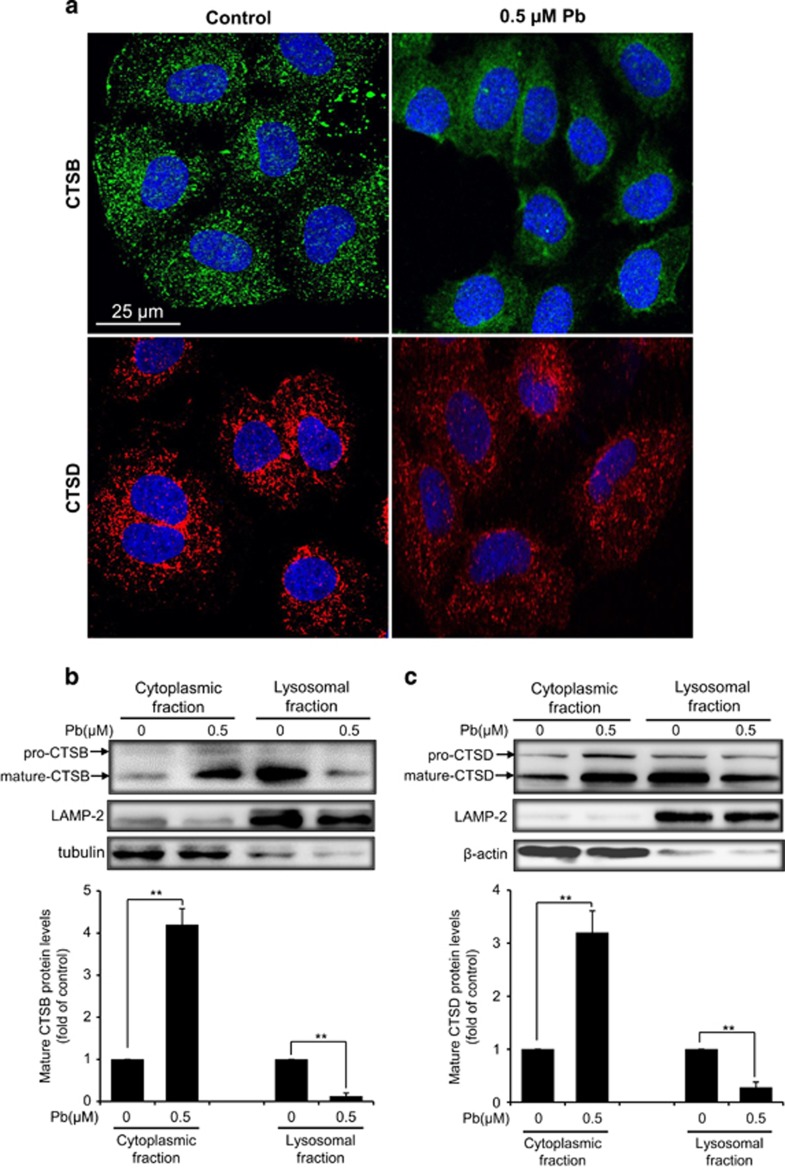
Release of CTSB and CTSD from the lysosome to the cytoplasm in Pb-exposed rPT cells. (**a**) Cells were treated with 0.5 *μ*M Pb for 12 h, then immunostained with antibodies against CTSB (green) and CTSD (red), respectively, and finally stained with DAPI (blue). Images were obtained by confocal microscopy. (**b** and **c**) Cells were treated with 0.5 *μ*M Pb for 12 h, then fractionated into the cytosolic and lysosomal lysates. CTSB (**b**) and CTSD (**c**) protein levels in different fractions were assessed by western blot analysis. Upper panels of **b** and **c** are representative western blot images and lower panels are quantitative analysis of protein levels (mean±S.E.M., *n*=4), respectively. **P<0.01

Decreased LTR fluorescence could also reflect LMP besides as an indicator of elevated lysosomal pH.^22^ To further determine whether LMP actually occurred in Pb-exposed rPT cells, two methods were conducted to verify this idea. Firstly, immunofluorescence analysis revealed the punctate staining of CTSB and CTSD in control cells, typical of a lysosomal distribution, while diffuse cytoplasmic CTSB and CTSD immunostaining was observed in Pb-exposed cells (Figure 7a). The release of lysosomal CTSB and CTSD into the cytoplasm demonstrated the occurrence of LMP. Secondly, the localization of CTSB and CTSD was analyzed by western blotting. Compared with the control group, Pb caused a decrease in CTSB and CTSD in the lysosomal fraction and a concomitant increase in the cytoplasmic fraction (Figures 7b and c), further confirming the permeabilization of lysosomal membranes following lead treatment.

### Pb-induced apoptosis can be alleviated by two cathepsin inhibitors in rPT cells

**Figure 8:**
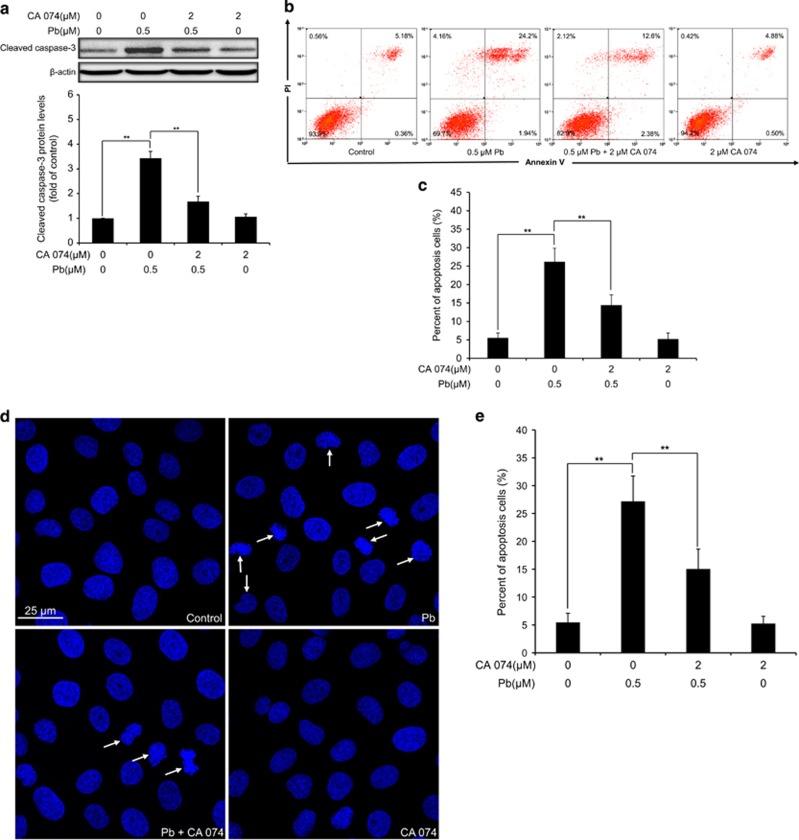
Pharmacological inhibition of CTSB activity prevented caspase-3 activation and apoptosis in Pb-exposed cells. Cells were pre-incubated with 2 *μ*M CA 074 for 1 h, then exposed to 0.5 *μ*M Pb for another 12 h to analyze the following assays. (**a**) Protein level of cleaved caspase-3 was assessed by western blot. Upper panel: representative western blot image; lower panel: quantitative analysis (mean±S.E.M., *n*=4). (**b** and **c**) Apoptosis was analyzed by flow cytometry. Representative flow cytometry analysis of Annexin V-PI staining is shown in (**b**) and its quantitative results (**c**) are expressed as mean±S.E.M. (*n*=9). (**d** and **e**) Apoptotic morphological changes were assessed by DAPI staining. Representative morphological changes of apoptosis are present in (**d**), and its statistical result of apoptotic rates (**e**) are expressed as mean±S.E.M. (*n*=9). **P<0.01

**Figure 9:**
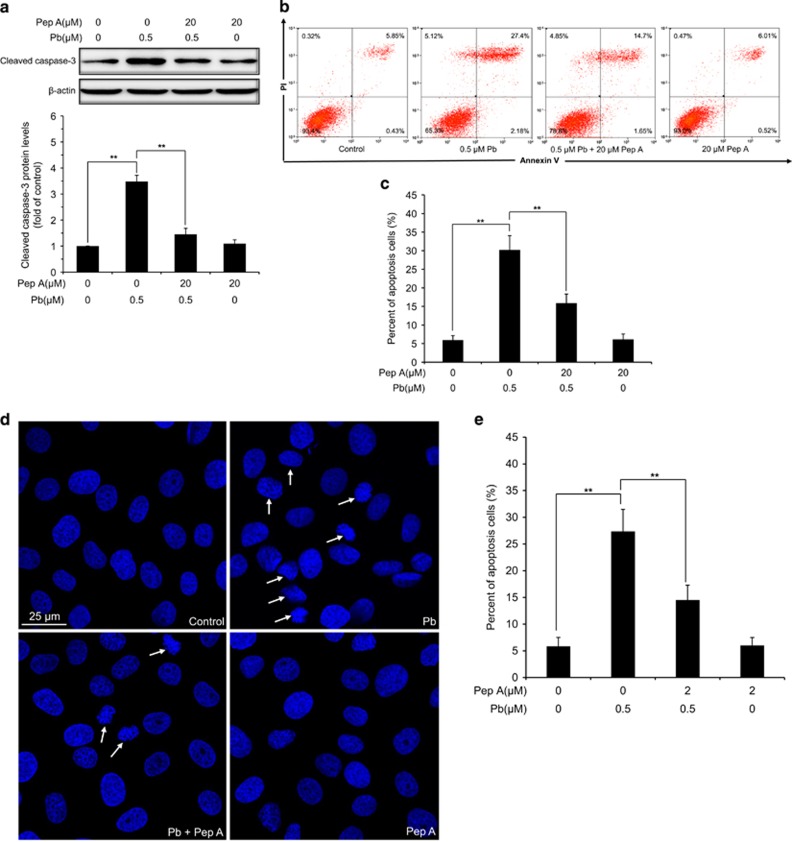
Pharmacological inhibition of CTSD activity alleviated Pb-induced caspase-3 activation and apoptosis in rPT cells. Cells were pre-incubated with 20 *μ*M Pep A for 1 h, then exposed to 0.5 *μ*M Pb for another 12 h to determine the following assays. (**a**) Protein level of cleaved caspase-3 was assessed by western blot. Upper panel: Representative western blot image; lower panel: quantitative analysis (mean±S.E.M., *n*=4). (**b** and **c**) Apoptosis was analyzed by flow cytometry. Representative flow cytometry analysis of Annexin V-PI staining is shown in (**b**) and its quantitative results (**c**) are expressed as mean±S.E.M. (*n*=9). (**d** and **e**) Apoptotic morphological changes were assessed by DAPI staining. Representative morphological changes of apoptosis are present in (**d**), and its statistical result of apoptotic rates (**e**) are expressed as mean±S.E.M. (*n*=9). **P<0.01

It is known that cathepsins released from the lysosomal lumen could activate apoptotic effectors such as mitochondria and/or caspases to trigger apoptotic cell death.^22^ To investigate whether LMP was involved in Pb-induced apoptosis in rPT cells, cells were pre-incubated with 2 *μ*M CA 074 (CTSB inhibitor) or 20 *μ*M Pep A (CTSD inhibitor) following 12-h Pb treatment to verify this idea, respectively. We found that CA 074 or Pep A significantly prevented Pb-induced caspase-3 activation (elevated cleaved caspase-3 level), while CA 074 or Pep A alone had no effect on cleaved caspase-3 level (Figures 8a and 9a). Consistent with this result, Pb-induced apoptosis can be significantly inhibited by co-treatment with CA 074 or Pep A, respectively, measured by two methods (Figures 8b–d and Figures 9b–d). Collectively, these findings supported the notion that LMP-induced lysosomal enzyme release was involved in Pb-induced apoptosis in rPT cells.

## Discussion

Recent studies have suggested that autophagy acts as a protective mechanism to promote cell survival during acute kidney injury, and blockade of autophagic flux has a negative impact on the development of nephrotoxicity.^7, 24, 25, 26, 27^ Our previous study identified that Pb exposure inhibited the autophagic flux in rPT cells,^12^ but the detailed inhibitory mechanism and the role of autophagy in Pb-induced nephrotoxicity are still unclear. Herein, the present experiments provide the first insight into the cellular mechanisms that the impairment of autophagic flux in Pb-exposed rPT cells was due to the compromised lysosomal function. We also found that Pb-induced lysosomal membrane permeabilization, leading to the release of cathepsins and consequently the initiation of apoptosis in rPT cells.

Autophagy is a dynamic process involving formation of autophagosomes (early stage) and lysosomal degradation of autophagosomes (late stage), which is tightly controlled by a group of autophagy-related (Atg) genes.^5^ Microtubule-associated protein light chain 3 (LC3), a mammalian homolog of yeast Atg8, is known to exist on autophagosomes, while the conversion of LC3 from the free form (LC3-I) to the phosphatidylethanolamine-conjugated form (LC3-II) represents a key step in autophagosome formation, and thus the net amount of cytosolic LC3-II is a critical hallmark for monitoring autophagy in mammalian cells.^5^ In this study, immunoblot analysis of LC3-II was first chosen to reflect the progression of autophagy in Pb-exposed rPT cells. As shown in Figure 1, Pb exposure elevated the LC3-II protein levels in rPT cells, indicating the possibility of enhanced autophagic activity. However, LC3-II accumulation can result from either autophagy activation or blockade of autophagic flux. To distinguish these two possibilities, pharmacologic agents targeting autophagy machinery (CQ and 3-MA) were applied in this study. GFP-LC3 transient transfection assay and western blot analysis of LC3-II revealed that Pb did not stimulate autophagy in rPT cells (Figures 2a–c).

To further investigate the effect of Pb on autophagy status, we monitored the total amount of p62 in rPT cells. p62 is a multifunctional protein that binds to LC3 and was degraded within the autolysosome, that is, enhanced p62 protein level has been regarded as an indicator for blockage of autophagic flux.^28^ Thus, changes of p62 protein levels (Figure 2d) gave us a hint that blockade of autophagic flux resulted in the accumulation of autophagosomes in this process. Consistent with our results, other heavy metals like arsenic and cadmium have been shown to block the autophagic flux in kidney of female mice and mouse neuroblastoma cells, respectively.^27, 29^ We further confirmed this notion with an RFP-GFP-LC3 construct, which is dependent on the fusion of autophagosomes with lysosomes, lysosomal acidification and degradation capacity.^30^ Data in Figure 2e indicated that the autophagic flux in Pb-treated rPT cells might be compromised most likely due to either defective fusion of autophagosomes with lysosomes or impaired lysosomal function. Moreover, fusion of autophagosomes with lysosomes to form autolysosomes is critical for autophagic flux and impairment of autophagosome–lysosome fusion will inhibit the degradation of autophagosomes.^31, 32^ In the present study, it showed that Pb did not block the fusion between autophagosomes and lysosomes because the endogenous LC3 protein was well colocalized with LAMP-1-positive compartments (Figure 3). Herein, this study was designed to investigate the potential role of lysosomal dysfunction in the course of Pb-induced autophagy inhibition.

One unique feature of lysosome is its highly acidic pH (4.5–5.0) that provides an optimal condition for its hydrolytic enzymes to perform their catalytic function.^33^ Lysosomal pH gradient is partly generated and maintained by V-ATPase, a multisubunit protein complex that hydrolyzes ATP to pump protons into the lysosome lumen.^34, 35^ Among the V-ATPases, ATP6V1A (V-ATPase subunit A) pumps H^+^ into the lumen of the organelles, having an important role in the acidification of lysosomal compartments.^36^ The 56-kDa “B” subunit is consisted of two highly homologous isoforms: ATP6V1B1 and ATP6V1B2, and these two subunits also have a part in organelle acidification.^37^ In addition, ATP6V1D is a subunit of a key enzyme for endosomal fusion and acidification of intracellular organelles.^38^ Here, we attempted to determine the possibility that Pb-induced autophagosome accumulation is due to disturbance of lysosomal internal environment. In this study, enhanced lysosomal pH in Pb-exposed rPT cells was clearly demonstrated by LTR and AO staining (Figure 4). Next, we examined whether Pb perturbs lysosomal pH by inhibiting V-ATPases activity. Data in Figure 5 showed that Pb significantly suppressed the protein levels of ATP6V1A, ATP6V1B1+ATP6V1B2 in rPT cells, but not affect the ATP6V1D protein levels. We hypothesize that inhibition of V-ATPase subunit A and subunit B may have an important role in the regulation of lysosomal acidification in Pb-exposed rPT cells. The detailed mechanism of Pb-induced lysosomal alkalinization in rPT cells is worthy of further investigation.

It is known that lysosomal proteolytic activity is dependent on acidic pH, which prompted us to further ascertain the proteolytic capabilities of lysosomes in Pb-exposed rPT cells. We did find that Pb inhibited lysosome-specific degradation with the DQ-BSA assay (Figure 6a). Cathepsins represent the largest group of proteolytic enzymes in the lysosome, wherein CTSB and CTSD are two abundant lysosomal proteases.^23, 39^ Since the maturation (activation) of cathepsin proteases requires acidification, the altered pH ultimately resulted in greatly reduced protein degradation.^40^ As shown in Figure 6b, Pb interfered with the maturation of CTSB and CTSD in rPT cells, further verifying that Pb inhibited the lysosomal function by affecting the maturation of selected proteases.

Furthermore, it is important to note that LMP is a critical step in the lysosomal cell death.^41, 42^ The distinctive sign of LMP is the translocation of soluble lysosomal components (including enzymes) from the lysosomal lumen to the cytosol.^20^ In this study, Pb-induced LMP was monitored by a variety of methods. It is reported that reduced red fluorescence and increased green fluorescence in AO-loaded cells together with decreased LTR fluorescence were used to reflect LMP.^20^ Given this, data in Figure 4 manifest the induction of LMP in Pb-exposed cells. Meanwhile, Pb induced the translocation of CTSB and CTSD from lysosomes to the cytosol, assessed by immunofluorescence and immunoblot assays (Figure 7), which provide solid evidence for LMP after Pb exposure. Due to their high hydrolase content, leakage of lysosomal content to the cytosol is potentially harmful to the cell. Partial permeabilization of the membrane induces apoptosis while massive lysosomal rupture induces necrosis.^13^ Once into the cytosol, CTSB and CTSD are the major mediators triggering apoptotic pathways, which involve Bid truncation, caspase activation and subsequent mitochondrial apoptosis.^20, 43, 44, 45^ To further validate whether cytosolic location of CTSB and CTSD are actively involved in Pb-induced apoptosis, rPT cells was pretreated with specific cathepsin inhibitors, that is, CA 074 and Pep A, to block the activities of CTSB and CTSD, respectively. In this study, Pb-induced caspase-3 activation and consequent elevated apoptosis were significantly but not completely prevented by the addition of CA 074 and Pep A, respectively (Figures 8 and Figures9). Collectively, these data indicate that Pb-induced LMP with partial release of the lysosomal content, that is, CTSB and CTSD, have an important role in apoptosis of rPT cells, but its detailed mechanism remains to be further clarified.

**Figure 10:**
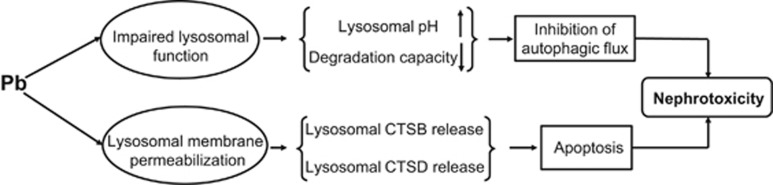
Scheme of the pathway of Pb-induced nephrotoxicity in rPT cells via impairment of autophagic flux and LMP-mediated apoptosis

In summary, the possible mechanism of Pb-induced nephrotoxicity in rPT cells via impairment of lysosomal function and induction of lysosomal membrane permeabilization is expounded (Figure 10). Firstly, impaired lysosomal function in Pb-exposed rPT cells is evidenced by lysosomal alkalinization and decreased lysosomal degradation capacity, which subsequently leads to the blockage of autophagic flux. Secondly, Pb-induced lysosomal membrane permeabilization results in the release of CTSB and CTSD from the lysosome to the cytosol, which triggers apoptotic cell death. Both autophagy blockage and LMP-mediated apoptosis contribute to Pb-induced nephrotoxicity in rPT cells. These findings will shed new light on understanding the molecular mechanism of Pb-induced nephrotoxicity.

## Materials and methods

### Chemicals and antibodies

All chemicals were of highest grade purity available. Lead acetate (PbAc_2_), collagenase IV, trypsin, 4′, 6-Diamidine-2′-phenylindole dihydrochloride (DAPI), antibiotic-antimycotic solution, DMEM-F_12_ (1:1), propidium iodide (PI), 3-methyladenine (3-MA, M9281), chloroquine diphosphate salt (CQ, C6628), acridine orange (AO, A6014), Earle’s Balanced Salt Solution (EBSS, E2888) and Lysosome Isolation Kit were purchased from Sigma-Aldrich (St. Louis, MO, USA). BCA protein assay kit and enhanced chemiluminescence (ECL) kit were obtained from Pierce Biotechnology (Rockford, IL, USA). Accutase cell detachment solution and Annexin V-FITC apoptosis detection kit were purchased from Pharmingen (Becton Dickinson Company, CA, USA). The CTSB inhibitor, CA 074, was obtained from Tocris Bioscience (Bristol, UK) and pepstatin A (Pep A, an inhibitor of CTSD) was purchased from Selleck chemicals, USA. Lipofectamine 3000 Transfection Reagent (L3000015), thapsigargin (TG, T7459), self-quenched bodipy-conjugated BSA (DQ-BSA-Green) (D-12050) and Lyso-Tracker Deep Red (LTR) (L12492) were purchased from Invitrogen (Carlsbad, CA, USA). The following antibodies were used: anti-LC3B (Sigma, L7543), anti-p62 (Sigma, P0067), anti-LAMP1 (Santa Cruz Biotechnology, Santa Cruz, CA, USA, sc-20011), Anti-ATP6V1A (Abcam, Cambridge, UK, ab199326), Anti-ATP6V1B1+ATP6V1B2 (Abcam, ab200839), Anti-ATP6V1D (Abcam, ab157458), anti-cathepsin B (CTSB, Santa Cruz Biotechnology, sc-13985), anti-cathepsin D (CTSD, Santa Cruz Biotechnology, sc-6486), anti-LAMP-2 (Sigma, L0668), anti-cleaved caspase-3 (Cell Signaling Technology, Danvers, MA, USA, 9664), anti-*α*-tubulin (Sigma, T6199) and anti-*β*-actin (Sigma, A5441). Secondary antibodies for western blotting analysis were conjugated to horseradish peroxidase (Jackson Immuno Research, West Grove, PA, USA, 705-505-303 and 111-006-062). Alexa Fluor 488-conjugated donkey anti-rabbit (ab150073), Alexa Fluor 555-conjugated goat anti-mouse (ab150114) and Alexa Fluor 555-conjugated donkey anti-goat (ab150134) secondary antibodies were purchased from Abcam.

### Cell culture and treatment

All procedures followed the ethics guidelines and were approved by the Animal Care and Use Committee of Shandong Agricultural University. Isolation, identification and culture of Sprague-Dawley rat proximal tubular (rPT) cells were as previously described.^46^ PbAc_2_, 3-MA and CQ were dissolved in sterile ultrapure water. CA 074 and Pep A were dissolved in DMSO to make the stock solution, filtered and stored at −20 °C, then diluted to work solution before use. The final concentration of DMSO was less than 0.1% which exhibits no effect on cell viability. Based on the doses of Pb in our previous study,^15^ rPT cells were incubated in the presence of 0, 0.25, 0.5 and 1 *μ*M Pb. Primarily, events of 0.5 *μ*M Pb exposure over a 12-h period were chosen to investigate the lead nephrotoxicity.

### Preparation of samples for immunoblotting

After the indicated treatments, cells were lysed in RIPA buffer (50 mM Tris-HCl pH 8.0, 150 mM NaCl, 0.1% SDS, 0.5% sodium deoxycholate and 1% NP-40) supplemented with protease inhibitors cocktail (Merck Millipore, Darmstadt, Germany) to prepare the whole-cell lysates. Moreover, cells subjected to designated treatments were collected and pooled together for lysosome isolation. Lysosomal fractions were extracted from cell homogenates by differential centrifugation followed by density centrifugation according to the manufacturer’s protocol (Lysosome Extraction Kit; Sigma-Aldrich; LYSISO1). Briefly, cell homogenates were centrifuged at 1000 × *g* for 10 min at 4 °C, then the supernatant fraction was centrifuged at 20 000 × *g* for 20 min at 4 °C. The resulting supernatant fraction was collected as cytosolic fraction and the pellet contains lysosomes and other organelles (crude lysosomal fraction). The pellet fractions were subjected to additional centrifugation (150 000 × *g* for 4 h at 4 °C). The final pellet (lysosomal) fraction was lysed in the lysis buffer described in the procedure for western blotting.

### Western blotting analysis

After protein quantification with BCA protein assay kit, samples were subjected to SDS-PAGE gels and transferred to PVDF membranes. After blocking with 5% skim milk for 1 h at room temperature, membranes were incubated overnight at 4 °C with the following primary antibodies: LC3B (diluted 1:1000), p62 (diluted 1:1000), LAMP-2 (diluted 1:400), CTSB (diluted 1:150), CTSD (diluted 1:150), cleaved caspase-3 (diluted 1:1000), ATP6V1A (diluted 1:2000), ATP6V1B1 + ATP6V1B2 (diluted 1:1000), ATP6V1D (diluted 1:10 000), *α*-tubulin (diluted 1:1000) and *β*-actin (diluted 1:5000). After several washes with TBST, the membranes were incubated with appropriate secondary antibodies (1:5000 dilution) for 1 h at room temperature. Finally protein bands were detected on a Chemidoc XRS (Bio-Rad, Marnes-la-Coquette, France) by using the ECL Kit. Proteins levels were then determined by computer-assisted densitometric analysis (Densitometer, GS-800, Bio-Rad Quantity One). The density of each band was normalized to its respective loading control (*β*-actin or *α*-tubulin or LAMP-2). Data obtained were expressed as the ratio of intensity of the protein in chemical-treated cells to that of the corresponding protein in control cells. Each test was performed in four experiments with different batches of cells.

### Plasmids and transient transfection

GFP-LC3 plasmid and RFP-GFP-LC3 plasmid were kind gifts of Dr. Xiao-Ming Yin (Department of Pathology and Laboratory Medicine, Indiana University School of Medicine, Indianapolis, Indiana, USA). Cultures of rPT cells at 60 to 80% confluence were transiently transfected with empty vector, GFP-LC3, or RFP-GFP-LC3 plasmids using Lipofectamine 3000 (Invitrogen, Carlsbad, CA, USA) according to the manufacture's protocol. After the indicated treatments, cells on coverslips were fixed with 4% paraformaldehyde (PFA) for 8 min at room temperature, then visualized by the confocal microscope (TCS SPE, Leica, Germany). Representative cells were selected and photographed. The number of puncta per cell was quantified using the ‘analyse particles’ function of ImageJ under identical threshold conditions.

### Assessment the fusion of autophagosomes and lysosomes

Cells were seeded on sterile coverslips placed in 24-well plates. After incubated with 0.5 *μ*M Pb (12 h) or 1 *μ*M TG (12 h) or EBSS medium (2 h), respectively, cell were fixed with 4% PFA for 8 min, permeabilized with 0.1% Triton X-100 in PBS for 15 min and blocked with 2% bovine serum albumin in PBS for 1 h at room temperature. Slides were first stained with anti-LC3 antibody (1:150 diluted in PBS) at 4 °C overnight. After washing the cells with PBS, cells were incubated with Alexa Fluor 488-conjugated donkey anti-rabbit secondary antibody (1:600 diluted in PBS) for 1 h at room temperature and washed with PBS again. Subsequently, cells were stained with anti-LAMP-1 antibody (1:80 diluted in PBS) at 4 °C overnight, washed with PBS again and incubated with Alexa Fluor 555-conjugated goat anti-mouse secondary antibody (1:500 diluted in PBS) for 1 h at room temperature. Nuclei were stained with DAPI (blue). Finally, all slides were mounted with ProLong Gold Antifade Mountant. Images were conducted on the Leica TCS SPE confocal microscope with a × 63 (1.3 numerical aperture) oil-immersion objective.

### Location of CTSB and CTSD by immunofluorescent staining

Cells were seeded on sterile coverslips placed in 24-well plates. After a 12-h incubation with 0.5 *μ*M Pb, cells were fixed with 4% PFA for 8 min, permeabilized with 0.1% Triton X-100 in PBS for 15 min and blocked with 2% bovine serum albumin in PBS for 1 h at room temperature. Then slides were stained with CTSB antibody (1:100 diluted in PBS) or CTSD antibody (1:100 diluted in PBS) at 4 °C overnight, respectively. After washing out the primary antibodies with PBS, cells were incubated with Alexa Fluor 488-conjugated donkey anti-rabbit secondary antibody (1:600 diluted in PBS) or Alexa Fluor 555-conjugated donkey anti-goat secondary antibody (1:500 diluted in PBS) for 1 h at room temperature, respectively. After removal secondary antibodies, nuclei were stained with DAPI (blue). Slides were mounted with ProLong Gold Antifade Mountant. Images were captured by a laser scanning confocal microscope (TCS SPE, Leica, Germany).

### Acridine orange staining

Acridine orange (AO), a lysosomotropic weak base that accumulates in intracellular acidic vesicles due to proton trapping, is usually used to measure the functional state of lysosomes.^20^ It is also a concentration dependent meta-chromatic fluorescent dye. Upon the excitation by blue light, AO can be visualized as red fluorescence at high concentrations (in intact lysosomes) and green fluorescence at low concentrations (in the cytosol and the nucleus). Thus, AO relocation, from lysosomes to cytosol, and the decrease of granular (lysosomal) red fluorescence (dimer form) in combination with the increased diffuse (cytosolic) green fluorescence (monomer form) may imply the deterioration of lysosomal membrane stability with a decreased proton gradient, which permits the leakage of the lysosomal contents to cytosol.^20^ After incubated with 0.5 *μ*M Pb (12 h) or 50 *μ*M CQ (3 h) or EBSS medium (2 h), respectively, cells grown on coverslips in 24-well plates were loaded with 5 *μ*g/ml AO at 37 °C for 30 min, rinsed twice with warm (37 °C) PBS and examined under confocal laser scanning microscope (TCS SPE, Leica, Germany) with excitation at 488 nm. Green fluorescence (emission peak between 530 and 550 nm) and red fluorescence (emission peak at about 650 nm) were simultaneously collected by two separate windows.

### Lyso-Tracker Red staining

Cells were seeded on sterile coverslips placed in 24-well plates. After treatment with 0.5 *μ*M Pb (12 h), EBSS medium (2 h) or 50 *μ*M CQ (3 h), respectively, cells were incubated with 100 nM LTR (diluted in DMEM-F_12_ medium) for 30 min under ideal growth conditions (37 °C, 5% CO_2_) to label the lysosomes. Then slides were rapidly washed with warm PBS (37 °C) for three times, mounted as described above and observed under a laser scanning confocal microscope (TCS SPE, Leica, Germany).

### Analysis of lysosomal degradation capacity

DQ-BSA-Green was used to determine the lysosomal degradation capacity. Cells grown on coverslips in 24-well plates were incubated with 10 *μ*g/ml of DQ-BSA-Green for 12 h (37 °C, 5% CO_2_), washed twice with PBS to remove excess probe and refreshed the medium. Then cells were treated with 0.5 *μ*M Pb (12 h), EBSS medium (2 h) or 50 *μ*M CQ (3 h), respectively. Slides were mounted and observed under a laser scanning confocal microscope with excitation set at 488 nm. Degradation capacity was measured by the green fluorescence signal released due to the degradation of DQ-BSA-Green.

### Analysis of apoptosis by morphological changes and flow cytometry

Cells were pretreated with 2 *μ*M CA 074 or 20 *μ*M Pep A for 1 h, followed by 0.5 *μ*M Pb treatment for another 12 h to assess its effect on Pb-induced apoptosis, respectively. Firstly, harvested cells under the indicated treatments were stained with annexin V/PI to analyze the distribution of apoptotic cells using flow cytometer. Secondly, DAPI staining was applied to assess the morphological changes of treated cells and 200 cells were randomly selected to count the apoptotic cells within every batch of experiment, each one performed in triplicate. Both of these two methods have been extensively described in our previous study.^15^

### Data presentation

Experiments were performed at least three times with similar results. Data are presented as the mean±S.E.M. of the indicated number of replicates. Statistical comparisons were made using one-way analysis of variance (ANOVA) (Scheffe’s *F* test) after ascertaining the homogeneity of variance between the treatments, and *P*<0.05 was regarded as significant.
